# An Overview of Fluvoxamine and its Use in SARS-CoV-2 Treatment

**DOI:** 10.7759/cureus.34158

**Published:** 2023-01-24

**Authors:** Naif A Arishi, Naif M Althomali, Ibrahim M Dighriri, Mohammed S Alharthi, Ghadeer B Alqurashi, Razan A Musharraf, Aeshah H Albuhayri, Marwah k Almalki, Shatha A Alnami, Zamzam O Mashraqi

**Affiliations:** 1 Department of Pharmacy, Qassim Armed Forces Hospital, Buraydah, SAU; 2 Department of Pharmacy, King Faisal Specialist Hospital and Research Center, Jeddah, SAU; 3 Department of Pharmacy, King Abdulaziz Specialist Hospital, Taif, SAU; 4 Department of Pharmacy, Taif University, Taif, SAU; 5 Department of Pharmacy, King Salman Bin Abdulaziz Medical City, Medina, SAU; 6 Department of Pharmacy, University of Tabuk, Tabuk, SAU; 7 Medical School, Alrayan Medical College, Medina, SAU; 8 Department of Pharmacy, Al-Emis Hospital, Jazan, SAU; 9 Department of Pharmacy, Al Nahdi Medical Company, Jazan, SAU

**Keywords:** covid 19, sars-cov-2, depression, obsessive-compulsive disorder, fluvoxamine

## Abstract

Fluvoxamine (FLV) is a well-tolerated, widely accessible antidepressant of the selective serotonin reuptake inhibitor (SSRI) category. It was formerly used to reduce anxiety, obsessive-compulsive disorder, panic attacks, and depression. Severe acute respiratory syndrome coronavirus 2 (SARS-CoV-2) is an enclosed ribonucleic acid (RNA) virus with a positive-sense RNA genome that belongs to the Coronaviridae family. Infection with SARS-CoV-2 causes clinical deterioration, increased hospitalization, morbidity, and death. As a result, the purpose of this research was to review FLV and its use in the treatment of SARS-CoV-2. FLV is a potent sigma-1 receptor (S1R) agonist that modulates inflammation by reducing mast cell downregulation, cytokine production, platelet aggregation, interfering with endolysosomal viral transport, and delaying clinical deterioration. FLV treatment reduced the requirement for hospitalization in high-risk outpatients with early identified coronavirus disease 2019 (COVID-19), defined by detention in a COVID-19 emergency department or transfer to a tertiary hospital. In addition, FLV may reduce mortality and risk of hospital admission or death in patients with SARS-CoV-2. The most common adverse effect is nausea; other gastrointestinal symptoms, neurologic consequences, and suicidal thoughts may also occur. There is no evidence that FLV can treat children with SARS-CoV-2. Although FLV is not expected to increase the frequency of congenital abnormalities during pregnancy, this risk must be balanced with the potential benefit. More research is required to determine the effectiveness, dose, and mechanisms of action of FLV; however, FLV appears to offer significant promise as a safe and widely accessible drug that can be repurposed to reduce substantial morbidity and mortality due to SARS-CoV-2.

## Introduction and background

Fluvoxamine (FLV) is a commonly prescribed antidepressant of the selective serotonin reuptake inhibitor (SSRI) family that physicians have used since the 1980s to treat anxiety, obsessive-compulsive disorder, panic, and depression [1,2]. The medication has also been shown to affect the sigma-1 receptor (S1R) in the endoplasmic reticulum (ER), which acts as a calcium signaling pathway regulator [3,4]. In 2019, data from mouse model research revealed that S1R agonists could be therapeutically effective in treating inflammation and sepsis [5]. The other proposed mechanisms of action of FLV include reduced platelet accumulation, decreased mast cell degranulation, interference with end lysosomal viral trafficking, and elevated melatonin levels, all of which may have direct antiviral effects when combined [6,7]. The FLV agonist in S1R promotes neurite outgrowth produced by nerve growth factors in cells [8]. S1R is an ER chaperone protein that has anti-inflammatory effects [9]. FLV's anti-inflammatory properties are most likely due to its modulation of S1R, which affects both immediate and chronic immune responses [10]. S1R is also a critical controller of inflammation caused by the inositol-requiring enzyme 1α [5]. SSRIs modulate inflammatory cytokine activities and gene expression in cellular and animal models of inflammation [11]. Preclinical data have contributed to the concept that FLV may have a significant therapeutic benefit for the treatment of coronavirus disease 2019 (COVID-19) [4,7]. FLV's ability to suppress cytokine storms of COVID-19. The severity of COVID-19 is related to an increase in inflammatory mediators such as cytokines and chemokines [12,13]. These findings have piqued the curiosity of researchers about the possible therapeutic function of S1R agonists in COVID-19 [14,15]. This study aims to review FLV and its use in severe acute respiratory syndrome coronavirus 2 (SARS-CoV-2) treatment.

## Review

SARS-CoV-2 replication molecular mechanisms

SARS-CoV-2 is an encapsulated ribonucleic acid (RNA) virus with a single-stranded positive RNA genome that belongs to the Coronaviridae family. SARS-CoV-2 life cycle is highly complicated, with phases that are controlled in both space and time [16,17]. After adhesion to target host cell binding sites, the most important of which is the angiotensin-converting enzyme 2 (ACE 2), the spike protein undergoes a conformational change as a consequence of ACE 2 receptor engagement, which is then sub-cleaved by furin and intended proteases like transmembrane protease serine 2 and cathepsin L [18]. After extraction and passage into the cytoplasm, the main protease controls the proteolytic conversion of viral polyproteins into proteins. Viral proteases then degrade viral polyproteins into 16 non-structural proteins required for the transcription and reproduction of viruses [19]. Furthermore, several messenger RNA (mRNA), including nested negative sense RNA originating from termination genomic RNA transcription, were linked to host immune responses [20,21]. Structural proteins, along with viral RNA and other nonstructural and accessory proteins, are believed to construct replication complexes gathered in locations adjacent to ER and Golgi spaces [22]. The genomic RNA and nucleocapsids are then thought to be translocated to reactivated sites where structural glycoproteins are present before assembly, ER transportation, and lysosomal escape [23,24]. As with other coronaviruses, the replication cycle appears significant in the ER, hijacking ER stress responses to improve protein translation [24].

FLV mechanisms to inhibit the development of SARS-CoV-2

FLV was predicted to have antiviral effects in the context of SARS-CoV-2 through several pathways. The connection to S1R is of the utmost importance in this process. S1R functions as a chaperone protein that facilitates signaling between ER, mitochondria, and the nucleus and promotes the correct twisting of freshly produced proteins while minimizing the buildup of unfolded proteins, thus essential in cell life and controlling the flow of calcium ion (Ca2+) from the ER into mitochondria under cellular stress [3]. In stressful situations, such as viral infection, S1R overexpression protects cells by reducing ER and oxidative stress and counteracting pro-apoptotic signals by inducing various processes that improve cell survival [25]. S1R ligands are extensively investigated as possible therapeutics against viral infections due to their crucial function in alleviating cellular stress during viral infection and examined SARS-CoV-2. In hepatitis C virus (HCV) infection, for example, the colocalization of S1R with nonstructural proteins involved in viral replicase complexes was observed [26]. SARS-CoV-2 was shown to interact with S1R, hijacking the translation mechanism in cells and favoring viral protein production. Molecules that target sigma receptors, such as antipsychotic medications and antihistamines, have been discovered to have anti-SARS-CoV-2 actions [27]. In an unbiased cell culture experiment, FLV was shown to disrupt HCV replication [28]. Furthermore, SARS-CoV-2 takes advantage of the unfolded protein response (UPR) and autophagy as responses to ER stress. The S protein has been identified to control UPR, favoring replication and biosynthesis of virus proteins [29,30]. Therefore, FLV modulatory action on the ER stress response may inhibit SARS-CoV-2 replication [6].

Potential site of action of FLV in SARS-CoV-2

Inhibiting endosomal proteases and reducing endosomal pH are both detrimental to endosomal activity and transportation. Modifying end-lysosomal pH may impede the assembly of viral replication complexes and viral trafficking and growth [31]. FLV is a lysosomotropic drug. Therefore, these compounds' lysosomotropic and endolysosomal PH-modulating properties might be helpful in the setting of SARS-CoV-2. The enzyme acid sphingomyelinase (ASM) has a well-established history of being linked to infectious viruses. ASM catalysis is the hydrolysis of sphingomyelin to phosphorylcholine and ceramide, which is involved in various cellular processes ranging from cytoskeletal rearrangement to proliferation, stress response, signaling, and induction of apoptosis. Because ASM is primarily a lysosomal enzyme, proteolytic activation causes the transfer to the cell membranes and ceramide formation [32,33]. Ceramide is found in cell membranes and regulates their biophysical properties. It comprises a sphingosine core which might be modified after translation to compounds such as glycosphingolipids. Though ceramide and ceramide-based molecules have a role in the transmission of various viral diseases, including influenza or rotavirus, whether ceramide or its derivative plays a role in the attachment or internalization of virus parts has yet to be determined [34]. Receptor ACE 2 for SARS-CoV-2 was identified as being localized to lipid traps [35]. In vitro investigations revealed that inhibition of ASM, specifically changes in the ceramide composition of the lipid rafts, inhibited the viral infection of cells [36]. FLV accumulates in lysosomes, disrupting and attenuating ASM function [37,38].

Anti-inflammatory effect of FLV

FLV may protect against severe SARS-CoV-2 by down-regulating the inflammatory response generated by a viral infection, regardless of its impact on S1R. Modulation of macrophage immune activity was hypothesized as a potential mechanism of action [39,40]. At the same time, depression is thought by some to be an inflammatory disorder [41]. Nazimek et al. have evaluated the anti-inflammatory effects of antidepressants and the involvement of macrophages [42]. Ishima et al. discovered that FLV is the most potent S1R agonist among ten antidepressants evaluated in a study evaluating the affinity of numerous antidepressants for S1R in the brains of rats [43]. Rosen et al. discovered in a 2019 study the effectiveness of FLV in preventing septic shock in mice; only 9% of wild-type (WT) mice died after injection with lipopolysaccharide, which is known to rapidly induce pro-inflammatory cytokines in mice and humans, compared to 62% of sigma-1. Similar results were obtained after fecal slurry infection, which resulted in significantly higher S1R than WT, with the mice succumbing to septic shock and death (p-0.05). S1R versus WT mice survival rates were comparable in the presence of an inositol-requiring enzyme inhibitor, showing the possible molecular effects of S1R to reduce the inflammatory response [5,44]. Platelet dysregulation is evident in severe COVID-19, characterized by increased activation, sensitivity, and aggregation [45,46]. FLV blocks platelet serotonin uptake through the sodium-dependent serotonin transporter (SERT). Restricting serotonin uptake interferes with platelet stimulation and accumulation, which lengthens the amount of time it takes for a wound to bleed, while simultaneously reducing the number of neutrophils recruited and the level of inflammation [40,47]. Therefore, FLV may help in advanced SARS-CoV-2 by counteracting the hypercoagulable state of platelets [48,49]. Furthermore, SSRI decrease histamine emission from mast cells and protease-1 mRNA levels; this is significant since activated mast cells have been associated with pulmonary thrombosis and edema in lung samples from COVID-19 patients [6,44].

Clinical evidence for the use of FLV in SARS-CoV-2

In the last two years, multiple studies have been conducted to evaluate the efficacy of various SSRI medicines for those infected with COVID-19. Lenze et al. performed the first clinical experiment (STOP COVID) in the United States during the 2020 pandemic [50]. The method was double-blind, and the participants were community-based adults. Patients whose oxygen saturation levels were low (92%) and who suffered from additional serious conditions were excluded from the trial. The onset occurred within seven days. Participants received 100 mg of FLV or a placebo for 15 days; no particular antiviral drug was administered as part of the procedure. The primary outcome was a low saturation level (92%), or dyspnea. No patient receiving FLV met the main goal; however, six of the 72 placebo patients had shortness of breath or low oxygen levels. One person required the assistance of a ventilator, and no participants perished. Despite the high dose of FLV used in this experiment, a patient with no respiratory limitations was admitted to the hospital for dehydration. The treatment group had 11 other adverse events, while the placebo group experienced six severe adverse events and an increase in the number of cases of pneumonia and gastrointestinal distress. This experiment had some limitations, such as a limited number of participants, a restricted number of outcomes, and no lengthy follow-up; overall, the results were good and showed that FLV may be used to prevent the worsening of early infection symptoms [50].

Seftel et al. initiated a prospective cohort trial in which FLV was provided as a treatment option for people during a large epidemic in late 2020. Sixty-five patients accepted FLV, while 48 opted for observation alone, without any particular therapy offered. There were no significant variations in fundamental demographics. However, only 38% of the individuals who took FLV were symptom-free, compared to 58% of those in the control group. Thirty percent of participants had at least one chronic condition, and the average age was 42. There were no hospitalizations in the FLV group, compared to 12.5% in the observation group (p = 0.005), demonstrating that FLV was a highly beneficial medication. Two of the patients required critical care, and one died. Although the FLV group had more early symptoms, on Day 14, the participants were symptom-free. In contrast, 60% of participants in observation groups continued to have anxiety, forgetfulness, exhaustion, and headaches, among other things (p = 0.001). This study had obvious imitations as a small-scale, open-label, nonrandomized trial. Yet, it provides essential information about how FLV could be used in occupational settings in a real-world situation [51].

In Brazil, a phase III study with four treatment arms was designed to minimize the need for emergency care due to worsening COVID-19 and hospitalization. Adults with SARS-CoV-2 and severe symptoms like influenza for less than seven days, and at least one enhancement factor were randomized for 10 days of therapy: ivermectin, FLV, metformin, or placebo. In the month of April 2021, metformin was discontinued but FLV was kept, and on August 6, 2021, the study arms were halted due to FLV superiority, with 3323 individuals enrolled. Relative risk (RR) of hospital admission or observation in the emergency department for more than six hours was 0.68 for FLV vs. placebo in the intention-to-treat group. In addition, in a per-protocol analysis of people who took at least 80% of tablets, FLV was beneficial against deterioration and death, with RR of 0.34 for hospitalization and an odds ratio (OR) of 0.09 for mortality [52] (Table 1).

Other research

Lenze et al. initiated a new randomized controlled experiment called STOP COVID 2 to validate the findings of the previous trial. This experiment was conducted in the United States and Canada, with network enrollment and telemedicine visits for all study contacts throughout the country. Due to the small sample size and relatively young and healthy individuals, the preliminary study (STOP COVID) concluded that there were few cases of clinical deterioration in general, resulting in poor precision in estimating the effect size [50]. STOP COVID 2 sought to recruit 1100 individuals using the same eligibility criteria as in the previous experiment; In this trial, however, the sample was enriched by requiring participants to have one or more of the following risk factors: over 40 years of age, obese, diabetic, hypertensive, having heart disease, lung disease, and an immune condition. STOP COVID 2 stopped accepting new participants based on an overall lower rate of clinical deterioration than expected, the trial's dwindling number of participants, and their analysis of the trial's unblended interim findings thus far. There were no negative safety signals; however, the study was no longer projected to enroll the required number of participants due to the successful rollout of vaccines in the United States and Canada. Participants who had previously registered for the study had completed their allocated therapeutic doses and terminated their scheduled follow-up questionnaires at 15 and 90 days to evaluate short- and long-term secondary results. This experiment had the same duration and primary endpoint as the first experiment but with reduced doses: 100 mg twice daily of FLV or placebo instead of three times daily for 15 days. With enrolment ceased, the study will need a larger sample size and more statistical power to identify FLV's impact on the main outcome. However, this research contains a secondary endpoint for healthy functioning and symptoms evaluated using the Global Health Scale after 15 days and again at three months [53].

Bramante et al. started Stage 1 of the COVID-19-OUT trial. The first stage of the quadruple-blind experiment included 70 patients, with 1160 people scheduled for the fully enrolled trial. This experiment included: FLV alone, metformin alone, ivermectin alone, metformin plus FLV, or metformin plus ivermectin with a placebo. Participants 30-85 years of age were included if they were asymptomatic or had experienced symptoms for 45 ml/min in three days after SARS-CoV-2 infection. Primary outcome measures were decreased oxygenation at 14 days, use of the emergency department for COVID-19 symptoms, and evaluation of post-SARS-CoV-2 infection at six and 12 months [54].

An ongoing trial headed by Naggie et al. includes three experimental arms (ivermectin, FLV, and fluticasone), each with a placebo comparison arm. Participants and study teams were aware of the research drug to which they have been assigned and were aware of whether they were in treatment or placebo groups. Confirmation of SARS-CoV-2 infection in study participants aged 30 and older occurred within ten days of initial participant screening. In addition, on June 8, 2021, people who had two or more existing symptoms of SARS-CoV-2 infection started signing up for this study, with a primary completion date of December 2022. The leading outcome indicators for this experiment are the number of hospitalizations, deaths, and symptoms reported by patients within two weeks [55].

Another study of FLV and COVID-19 in Hungary compared the length of time it took for patients to show improvement when taking 100 mg of FLV twice a day vs. a placebo for 74 days. In this study, 100 patients with severe COVID-19 were included. The main objective of clinical recovery was the return to normality of three of four clinical markers listed below: temperature, respiratory rate, pulse oxygen saturation, and cough load [56]. The results of these ongoing trials are eagerly awaited for publication, as they will contribute significantly to our understanding of how and why early FLV therapy may benefit COVID-19-related morbidity and death (Table 1).

**Table 1:** Summary studies on FLV in SARS-CoV-2 RCT: randomized clinical trial; FLV: fluvoxamine; COVID-19: coronavirus disease 2019; SARS-CoV-2: severe acute respiratory syndrome coronavirus 2; RR: relative risk; OR: odd ratio

Authors and date	Design and sample size	Intervention and duration	Study population	Results or endpoints
Lenze et al., 2020 [50]	RCT; Sample size: 152	FLV 100 mg, 3 times a day for 14 days; duration: 15 days	Aged ≥ 18, SARS-CoV-2 infection not hospitalized identified < 7 days symptoms beginning	Within 15 days, no FLV individuals met the criteria for clinical deterioration, while 6 (8.3 %) placebo participants did.
Seftel et al., 2021 [51]	Observational cohort; sample size: 113	FLV 50–100 mg load, then 50 mg 2 times daily for 14 days; duration: 2 weeks	Workers in a crowded living environment during a COVID-19 epidemic had positive SARS-CoV-2 following mass testing.	FLV was administered to 65 patients; zero (0/65) had been hospitalized due to clinical deterioration, and 12.5% of those who refused therapy had been hospitalized (p = 0.005). At Week 2, 0% of individuals in the FLV group had continued COVID-19 symptoms, but 60% of those who did not receive therapy reported symptoms (p = 0.001). 21% reporting ≥ 5 signs at Week 2.
Reis et al., 2021 [52]	RCT; sample size: 3323	FLV 100 mg, 2 times daily for 100 days; duration: 28 days	In Brazil, positive SARS-CoV-2 test, aged > 18, flu symptoms for less than 7 days, and at least one enhancement factor	The percentage of patients with COVID-19 observed for > 6 h or transferred to a tertiary hospital was lower for FLV than for placebo (11% vs. 16%); RR = 0·68, Having a probability of 99.8 %, above the predetermined criterion of the superiority of 97.6%. Of the composite primary outcome events, 87% were hospitalizations. The intention-to-treat analysis yielded comparable results for the primary outcome (RR = 0·69) and more remarkable in the per-protocol study (RR = 0·34). In the main intention-to-treat analysis, there were 17 deaths in FLV and 25 deaths in placebo (OR= 0·68). In the per-protocol population, there was only one fatality in FLV and 12 in placebo (OR = 0·09). In addition, the FLV and placebo individuals had similar numbers of side events.
Lenze et al., 2022 [53]	RCT, Phase III; sample size: 1100	FLV 100 mg, 2 times a day for 14 days; duration: 15 days/3 months	USA and Canada, not hospitalized diagnosed SARS-CoV-2 infection, aged ≥ 18, one or more of the following risk factors: aged ≥ 40, diabetes, hypertension, obesity, heart disease, immune condition, and lung disease	The clinical worsening is the primary endpoint: dyspnea or hospitalization and hypoxia with < 92 % oxygen saturation.
Fekete et al., 2022 [56]	RCT, Phase II; sample size: 100	FLV 100 mg, 2 times daily for 74 days; duration: 74 days	Hungary; hospitalized, confirmed SARS-COV-2, aged 18 to 70, moderate symptoms, oxygen saturation ≥ 93%, and pneumonia	The primary outcome is the time to recovery after therapy, defined as having 3 of the 4 elements: Antipyretics can get rid of a fever for at least 48 hours, respiration rate ≤ 20/min, at room air oxygen saturation ≥ 95% and any decrease on the cough-burden, compared to baseline.
Bramante et al., 2022 [54]	RCT, Phase III: Sample size: 1160	FLV 50 mg, 2 times daily for 14 days; duration: 12 months	United States; aged 30 to 85, not symptomatic > 7 days after randomization, and glomerular filtration greater than 45 ml/min in two weeks for patients > 75 years or with liver failure, history of heart or renal failure	Primary endpoints include a 14-day reduction in oxygenation (oxygen saturation ≤ 93% on home surveillance) and 14-day emergency department usage for COVID-19 symptoms. Secondary outcomes include the maximum intensity of symptoms at 14 and 28 days, the period for significant healing, and a set of test results on Days 1, 5, and 10
Naggie et al., 2022 [55]	RCT, Phase III; sample size: 15,000	FLV 50 mg, 2 times daily for 10 days; duration: 29 days	United States; not hospitalized currently or within ten days of screening, or previously diagnosed with COVID-19 infection and aged ≥ 30 years. Must have SARS-CoV-2 Within 10 days after the screening, the condition was verified, and 2 or more current symptoms of acute infection persisted for at least seven days.	Primary endpoints include number, number of deaths, and number of, all within 2 weeks, according to patient reports. Secondary outcomes include Variation of the COVID clinical progression scale, number of hospitalizations in 28 days, number of deaths in 28 days, and count of resolutions of symptoms and hospitalizations as a total score.

FLV efficacy

Randomized trials have shown that FLV is effective in preventing the worsening of clinical status or development of chronic morbidity in patients infected with SARS-CoV-2.

 Data from the release of the TOGETHER study and the first STOP COVID study revealed the extent of FLV efficacy is unknown; in both the STOP COVID and TOGETHER studies, no participants received FLV; those infected with COVID-19 were more likely to have respiratory worsening and hospitalization than those in the control group (8.3 vs. 12.5%). Comparable results were shown in a per-protocol analysis of the TOGETHER research, where the FLV arm was linked to a 66% reduction in clinical deterioration requiring hospitalization or extended emergency contact and a 91% decline in mortality [43,52]. Nevertheless, STOP COVID discovered no variations in short-term symptomatic healing and noted lingering symptoms identified after four months in both the FLV and placebo, although the numbers were too small to allow statistical comparisons. The Settle study also found that no participants in the FLV group had continuous symptoms at 14 days, compared to 60% in the control group. Even though most of the media and public attention has been focused on COVID-19 deaths, SARS-CoV-2 infections cause both short-term and long-term illnesses, making them a major global health concern.

FLV Safety

FLV has a very good safety record, with nausea being the most prevalent side effect [57,58]. In the study of 35,368 people who took FLV in different countries, 15.7% reported nausea. This was followed by sleepiness (6.4%), fatigue (5.1%), and migraine (4.8%). Approximately 2.0% of people use this drug for mental illness, with 1.6% requiring hospitalization and 0.4% having another serious problem such as suicidality or distress. [57]. The most common side effect of FLV, when used to treat mental problems, is nausea, although additional gastrointestinal symptoms, neurologic outcomes, and occasionally suicidal thoughts can occur [59,60].

FLV drug-drug interactions

FLV is a substrate of CYP2D6, a potent inhibitor of CYP1A2 as well as CYP2C19. FLV can aggravate the serotonergic effects of other SSRIs, resulting in serotonin disorder; thus, FLV should not be taken within two weeks of another SSRI. In addition, FLV can increase the effect of antiplatelets and anticoagulants, so people taking these drugs should be watched closely [1,61,62].

Considerations in children

FLV is used to treat obsessive-compulsive disorder in children. The adverse consequences of use in young children are comparable to those reported in adults, even though children tend to have higher rates of behavior therapy and vomiting than adults [63,64]. There is no evidence of the use of FLV in children to treat SARS-CoV-2.

Considerations in pregnancy

Although FLV is not expected to increase the incidence of congenital defects, research on its use during pregnancy is limited [65,66]. In addition, although the absolute risk is likely minimal, the relationship between SSRI use in the late third trimester with a slight but elevated risk of pulmonary hypertension in infants has not been ruled out [67]. Therefore, the danger of using FLV during pregnancy to treat SARS-CoV-2 should be weighed against the possible benefit.

## Conclusions

FLV is a regularly prescribed antidepressant from the SSRI class used to treat anxiety, panic attacks, and depression. The SARS-CoV-2 infection leads to the deterioration of patients, prolonged hospitalization, morbidity, and mortality. Several studies have been conducted in the past two years to evaluate the effectiveness of different SSRI drugs for people infected with COVID-19. FLV was the main focus of clinical studies. FLV is a potent S1R agonist that modulates inflammation by reducing mast cell downregulation, cytokine production, platelet aggregation, interference with endolysosomal viral transport, and delayed clinical deterioration, all of which have been shown to have a strong antiviral effect, control coagulopathy, or alleviate the cytokine storm, all of which have been known cornerstones of extreme COVID-19. FLV treatment reduces the need for hospitalization in high-risk outpatients with an early diagnosis of COVID-19, as determined by admission to a COVID-19 emergency department or transfer to a tertiary hospital. In addition, FLV may marginally reduce all-cause mortality and the likelihood of hospitalization or death in outpatients with moderate COVID-19. The most common adverse effect of FLV is nausea; other gastrointestinal symptoms, neurologic consequences, and suicidal thoughts may also occur. FLV is a safe and readily available medicine that could lessen the severe morbidity and death associated with SARS-CoV-2. However, the findings of the ongoing studies are eagerly awaited since they will contribute significantly to our knowledge of how and why early FLV medication can help patients' morbidity and mortality associated with COVID-19.
